# Three for the Price of One: Concomitant I⋯N, I⋯O, and I⋯π Halogen Bonds in the Same Crystal Structure

**DOI:** 10.3390/molecules27217550

**Published:** 2022-11-03

**Authors:** Steven van Terwingen, Ruimin Wang, Ulli Englert

**Affiliations:** 1 Institute of Inorganic Chemistry, RWTH Aachen University, Landoltweg 1, 52074 Aachen, Germany; 2 Key Laboratory of Chemical Biology and Molecular Engineering of the Education Ministry, Shanxi University, 92 Wucheng Road, Taiyuan 030006, China

**Keywords:** halogen bond, QTAIM, energy density, pyrazole, electron density, hcb net

## Abstract

The ditopic molecule 3-(1,3,5-trimethyl-1*H*-4-pyrazolyl)pentane-2,4-dione (HacacMePz) combines two different Lewis basic sites. It forms a crystalline adduct with the popular halogen bond (XB) donor 2,3,5,6-tetrafluoro-1,4-diiodobenzene (TFDIB) with a HacacMePz:TFDIB ratio of 2:3. In a simplified picture, the topology of the adduct corresponds to a **hcb** net. In addition to the expected acetylacetone keto O and pyrazole N acceptor sites, a third and less common short contact to a TFDIB iodine is observed: The acceptor site is again the most electron-rich site of the pyrazole π-system. This iminic N atom is thus engaged as the acceptor in two orthogonal halogen bonds. Evaluation of the geometric results and of a single-point calculation agree with respect to the strength of the intermolecular contacts: The conventional N⋯I XB is the shortest (2.909(4) Å) and associated with the highest electron density (0.150 *e*Å${}^{-3}$) in the bond critical point (BCP), followed by the O⋯I contact (2.929(3) Å, 0.109 *e*Å${}^{-3}$), and the π contact (3.2157(3) Å, 0.075 *e*Å${}^{-3}$). If one accepts the idea of deducing interaction energies from energy densities at the BCP, the short contacts also follow this sequence. Two more criteria identify the short N⋯I contact as the most relevant: The associated C–I bond is significantly longer than the database average, and it is the only intermolecular interaction with a negative total energy density in the BCP.

## 1. Introduction

Halogen bonds (XBs) arise from a local electron deficiency of a (mostly heavy) halogen on the opposing site of its σ-bond [1]. This so-called *σ-hole* [2] can interact with Lewis bases to form XBs; suitable acceptors are N-heterocycles [3,4,5,6], halides [7,8,9], or even π-systems [3,10,11,12]. In their most common appearance with a single atom bonded to the heavy halogen, XBs exhibit approximately linear geometry; together with their strongly electrostatic nature, they are related to hydrogen bonds [13,14]. Although their first explicit observation occurred in 1954 by Hassel et al. [15], it was not until the turn of the millennium that halogen bonds attracted the broad attention of both the theoretical and experimental crystal engineering community. Several groups have made significant contributions to the theoretical description of XB interactions, but only a few can be mentioned here [16,17,18]. Since 2005, research on this topic has rapidly expanded; it has been reviewed several times [12,18,19,20] and has become omnipresent in crystal engineering.

**Figure 1:**
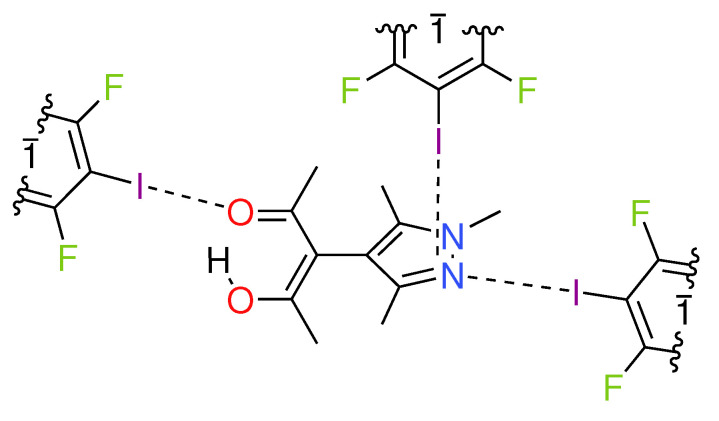
Chemical diagram of the asymmetric unit found in the crystal structure of HacacMePz·1.5TFDIB (**1**); inversion centers are marked with $\bar{1}$.

We have recently shown that the anion X^-^ from hydrohalides of substituted N-heterocycles may form both halogen and hydrogen bonds in the same solid [8]; only shortly after, the Cinčić group published multiple halogen and hydrogen bonded adducts of halopyridinium salts [9]. Herein, we report that the same substituted heterocycle can act as a multi-halogen bond acceptor, albeit without being protonated. In this solid, three different halogen bonds, namely I⋯N, I⋯O, and I⋯π, occur and can be compared directly. We decided for TFDIB as the XB donor, a particularly popular partner for XB-driven concrystallization: The CSD [21] comprises roughly 500 error-free entries in which a TFDIB iodine approaches an acceptor (N, O, Cl) at a distance shorter than the sum of the van der Waals radii. A chemical diagram of the asymmetric unit of our target cocrystal 3-(1,3,5-trimethyl-1*H*-4-pyrazolyl)pentane-2,4-dione (HacacMePz) with 1.5 equivalents of 2,3,5,6-tetrafluoro-1,4-diiodobenzene (TFDIB) **1** is given in Figure 1. We propose that all interactions shown in the figure may be exploited for crystal engineering purposes; similar motifs, where one molecule accepts multiple halogen bonds, have also been reported recently [22]. In addition to geometry arguments, which are based on crystallographic results, we corroborate our findings by a single-point calculation and subsequent analysis of the associated electron density according to Bader’s Quantum Theory of Atoms in Molecules (QTAIM) [23].

## 2. Materials and Methods

Searches in the Cambridge Structural Database [21] (CSD, version 5.43, including the update of September 2022) were restricted to perfluorinated iodobenzenes.

All chemicals were used as purchased without further purification. 3-(1,3,5-Trimethyl-1*H*-4-pyrazolyl)pentane-2,4-dione (HacacMePz) was synthesized as published before [24]. Single crystal X-ray intensity data was collected with a Bruker D8 goniometer equipped with an Incoatec microsource (Mo-K_α_ radiation, λ = 0.71073 Å, multilayer optics) and an APEX CCD area detector. A temperature of 100 K was maintained with an Oxford Cryostream 700 instrument, Oxfordshire, UK. Data was integrated with the Bruker SAINT program [25] and corrected for absorption by multiscan methods [26]. The structure was solved by intrinsic phasing [27] and refined with full matrix least squares procedures against $F^{2}$ [28]. Crystal data and refinement details are summarized in Table S1. The CIF for **1** has been deposited under CCDC No. 2209103. The powder X-ray diffraction pattern was recorded as a flat sample at room temperature with a STOE STADI-P diffractometer (Guinier geometry, Cu-K_α_ radiation, Johann Ge monochromator, STOE IP-PSD image plate detector, 0.005° 2θ step width). It shows that the bulk essentially corresponds to the phase established by single crystal diffraction (Supplementary Materials Figure S1). Thermogravimetric analysis (TGA) and differential scanning calorimetry (DSC) were performed using a Linseis STA PT 1600 instrument (Figure S2, Table S2). The sample was placed in a sealed Al_2_O_3_ crucible with a volume of 0.12 mL with a hole in the lid. Heating was applied at a rate of 5 K min^−1^ from room temperature to 500 °C under a constant flow of N_2_ with a flow rate of 60 mL min^−1^.

### 2.1. Synthesis and Crystallization

HacacMePz (10.4 mg, 0.05 mmol, 1.0 eq.) and TFDIB (30.1 mg, 0.075 mmol, 1.5 eq.) were dissolved in chloroform (2 mL). The solution was left unperturbed for slow evaporation at room temperature. After one week colorless, rod-shaped crystals formed. CHN: anal. calcd. for C_20_H_16_F_6_I_3_N_2_O_2_: C 29.6%, H 2.0%, N 3.5%; found: C 30.8%, H 2.2%, N 3.8%.

### 2.2. Computational Details

Before the single-point calculation was carried out, the C–H and O–H distances were idealized to values obtained from neutron diffraction experiments [29]. The theoretical electron density ρ was obtained from a single-point calculation of an expanded asymmetric unit (Figure S3) in the geometry established by X-ray diffraction; cartesian coordinates are available in the Supporting Information. The calculation was performed at the DFT level of theory with the M06-2X functional [30] and the MIDIX basis set [31] with the program Gaussian [32]. The derived electron density was analyzed with AIMAll [33] and Multiwfn [34] and interpreted with Bader’s QTAIM [23]. Additionally, the kinetic energy density *G* and its ratio with the electron density G/ρ in the bond critical points (BCPs) were derived as suggested by Abramov [35]. Furthermore, the potential energy density *V* was calculated using the local virial theorem [36,37]. The interaction energies of the short contacts were estimated, as suggested by Espinosa et al. [36], by the equation $E_{\mathrm{XB}}\approx 0.5V_{\mathrm{BCP}}$. $E_{\mathrm{tot}}$ was calculated with CrystalExplorer [38,39] with the “fast” setting (HF/3-21G level).

## 3. Results

### 3.1. Structural Features of ***1***

**Figure 2:**
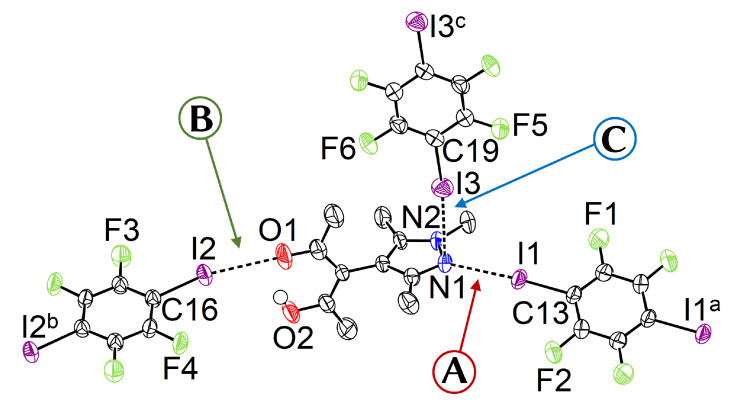
Displacement ellipsoid plot [40] of **1** with partial atom labeling (90% probability, carbon bonded hydrogen omitted). Selected distances and angles (Å, °): I1⋯N1 2.909(4), I2⋯O1 2.929(3), I3⋯Pz (Distance between I3 and the least squares plane of the pyrazole ring, consisting of the five atoms N1, N2, C7, C8, and C9) 3.2157(3), I1–C13 2.098(3), I2–C16 2.061(3), I3–C19 2.077(3), C13–I1⋯N1 172.05(12), C16–I2⋯O1 167.37(13), C19–I3⋯N1 173.35(12), ω 88.8(2). Symmetry operators: a = −x,1−y,−z; b = 3−x,1−y,2−z; c = −x,−y,1−z.

We first discuss the X-ray crystal structure of **1**. In order to achieve precise geometry data and account for the obviously large absorption in solid **1**, we collected data up to a high resolution of $\sin(\theta_{\max})/\lambda \approx$ 1.0 Å${}^{-1}$ with a redundancy of approximately 6.0, acceptably high for the triclinic symmetry. **1** crystallizes in space group $P\bar{1}$ with Z=2; a displacement ellipsoid plot with important distances and angles is given in Figure 2.

The angle ω between the least squares planes of the pyrazole and the acetylacetone moiety is close to 90°; this is expected and within the energetically favored range of possible ω angles [24]. There are three independent TFDIB moieties, all located on different inversion centers. In the following, their shortest contacts to the substituted pyrazole molecule are referred to as capital letters **A**, **B**, and **C** (Figure 2).

**A**  The pyrazole N⋯I halogen bond occurs between the TFDIB moiety located on Wyckoff position 1c and N1 at a N⋯I distance of 2.909(4) Å. For sufficiently precise data, anti-correlation between short I⋯donor XBs and long C–I bonds was reported [41]. Our data for **1** meets these requirements and allow us to discuss the competing XBs in the light of their associated C–I bonds. We found that C13–I1 is elongated and 0.02 Å is longer than the corresponding bond in pure TFDIB (CSD refcode ZZZAVM02 [42]). Only two contacts between a pyrazole and TFDIB were documented in the CSD; they amount to 2.860 Å in TOJBIE [43] and 2.934 Å in TIPKAH (In refcodes TIPKAH and AWUWOH, not *p*-TFDIB, but *o*-TFDIB was used) [44].
**B**  Another short contact exists between the acetylacetone keto O1 and I2 of the second TFDIB moiety, occupying the positions close to the inversion center with Wyckoff letter 1e; it amounts to 2.929(3) Å. As expected, the C–I bond in this moiety is less elongated than the C–I bond in the I⋯N halogen bond **A**. This is due to the weaker basicity of oxygen compared to the iminic nitrogen of the pyrazole moiety. For similar motifs, such as pyridyl substituted β-diketones, I⋯O_keto_ amounts to about 3.05 Å (refcodes TAXYID [45], AWUWOH^‡^ [46]). In all cases of protonated β-diketones, the halogen bond acceptor is the keto oxygen, not the enol bond acceptor. Chemical intuition suggests that the keto oxygen is associated with the more negative charge. In several cocrystals of β-diketonato complexes with TFDIB, two oxygens of different β-diketonate ligands act as halogen bond acceptors; the XB is oriented more or less symmetrically bifurcated towards the midpoint between these two oxygen atoms [47,48].
**C**  Last but not least, I3 from the third symmetry independent TFDIB moiety, located around the inversion center with Wyckoff position 1b, acts as XB donor towards the pyrazole π-system with a distance of 3.2157(3) Å. As expected by the theoretical electrostatic potential for pyrazoles [49], the closest contact atom for I3 is the iminic N1 with a distance of 3.241(4) Å. Lewis basic π-systems as XB acceptors are known in literature, e.g., for cyclopentadienyl ligands [50], imidazoles [51], or carbazoles [52], and have been evaluated theoretically [53,54,55]; however, to the best of our knowledge, no pyrazole-π⋯I interactions with perfluoronated iodobenzenes have been reported to this date. This is also due to the competition with the more prominent I⋯N XB, as present in interaction **A**.

**Figure 3:**
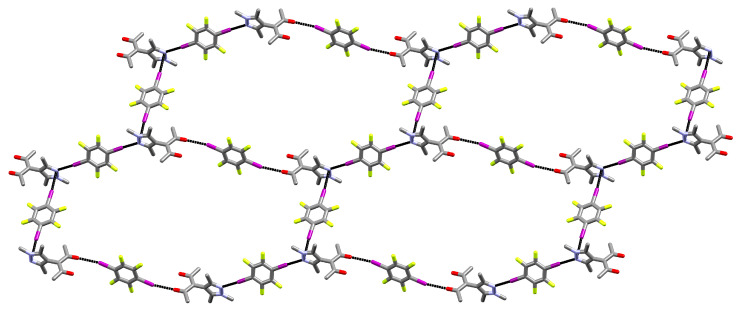
Excerpt of the two-dimensional net formed by the three halogen bonds towards one HacacMePz moiety in **1**, shown perpendicular to the (−2 3 3) plane (hydrogens omitted) [57].

If the halogen bonds **A** and **B** are taken into account, an extended 1D structure can be derived. This chain expands along [3 0 2] in a “zig-zag” manner. Adding the third halogen bond **C** (C–I⋯π_Pz_) to the contacts, a two-dimensional net can be perceived. It expands in the (–2 3 3) plane and no strict analogy can be found in the Reticular Chemistry Structure Resource (RCSR) [56]. If the N1 sites are perceived as triconnected vertices and the net is simplified by treating all edges as equivalent, its topology matches the honeycomb **hcb** net (Figure 3).

Differential scanning calorimetry and thermogravimetry of **1** show that the melting point of the XB acceptor roughly corresponds to the decomposition point of the adduct (Figure S2). This behavior is commonly encountered for XB adducts [58]. Afterwards, at around 112 ${}^{\circ }$C to 192 ${}^{\circ }$C, a continuous weight loss of 64% is observed, which roughly corresponds to the loss of one HacacMePz moiety, together with two equivalents of TFDIB (calcd. 62.4%), leaving a stoichiometry of 1:1. Over the next approximate of 200 ${}^{\circ }$C, further weight loss of 25% is observed, which corresponds to one TFDIB moiety (calcd. 24.8%).

### 3.2. Theoretical Electron Density Considerations

**Figure 4:**
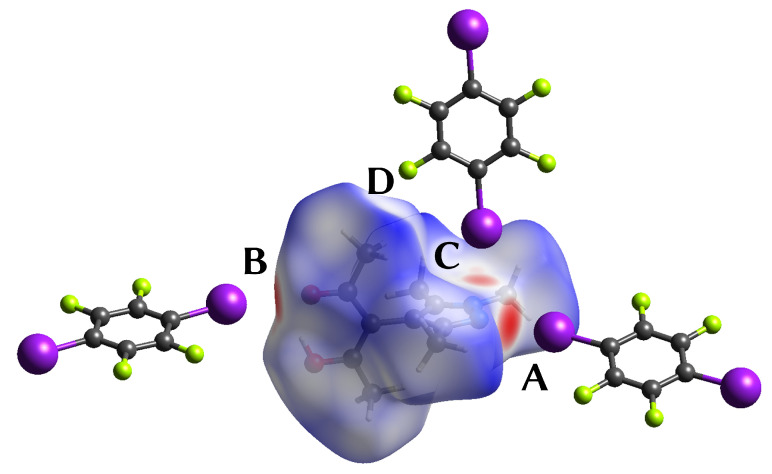
Depiction [38] of the Hirshfeld surface of the HacacMePz moiety mapped with $d_{\mathrm{norm}}$ (contacts **A** to **D** marked); regions marked in red represent close contacts.

When $d_{\mathrm{norm}}$ is mapped on the Hirshfeld surface [59] about the HacacMePz moiety in **1**, the halogen bonds show up as close contacts (Figure 4). Additionally, a rather close contact between a methyl group to a fluorine (**D**) was highlighted.

In addition to the geometry arguments mentioned above, further insight about the coexisting XBs in **1** may come from the electron density and its derived properties, such as energy densities. For this purpose, a single-point calculation was performed, and the resulting electron density was analyzed by Bader’s QTAIM [23]. Trajectory plots reveal that all contacts **A** to **D** are associated with essentially linear bond paths (Figures S4 and S5). In Table 1, characteristics of the aforementioned contacts in their BCPs are compiled.

We are not aware of charge density studies on halogen bonds to pyrazoles, but TFDIB represents a particular popular XB donor. The experimental electron density in its N⋯I contacts to other N heterocycles, such as pyridine [5,48,60] and O⋯I interactions to bipyridine oxide [61] and water [48], have been reported. Both N⋯I and O⋯I bonds involving TFDIB in the same crystal structure have been characterized by high resolution diffraction [48]; this study has found experimental electron densities, which closely match the outcome of the single point calculations for **A** and **B** reported here.

**Figure 5:**
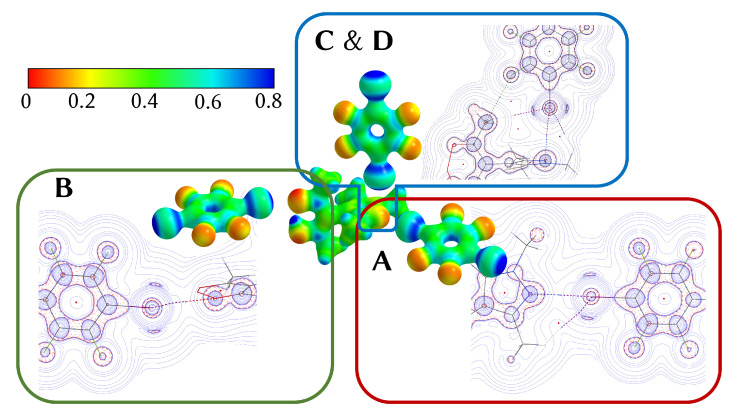
Center: Electrostatic potential of **1**, mapped onto an isosurface of ρ = 0.07 a.u. (scale given in the top left) [33]; excerpts of the Laplacians of contacts **A** to **D** are shown perpendicular to their respective TFDIB plane (contour lines drawn at $\pm 2^{n}\;\cdot \;10^{-3}$ a.u. with 0≤n≤20).

The electrostatic potential derived from the theoretical electron density offers an intuitive way to visualize XBs. Figure 5 shows the negative potential at the halogen acceptors **A** and **B** and the side-on interaction **C**. For each short contact the Laplacian of the electron density has been included.

It is an attractive and somewhat controversial [62] idea that intermolecular interaction energies might be directly deduced from properties of the electron density ρ in the BCP between the contact atoms. If one accepts this concept, ρ in the BCPs represents the first criterion to gauge the strength of such interactions. From the more conventional and stronger halogen bonds **A** and **B** over the perpendicular π-type contact **C** to the presumably weak interaction **D** between a fluorine and a methyl group with their opposite charges, the electron density in the BCPs decreases (Table 1). Additional insight may come from energy density considerations: The (positive) kinetic energy density *G* and the ratio $G/\rho_{\mathrm{bcp}}$ have been suggested as qualifiers for chemical bonding. When expressed in atomic units, the ratio $G/\rho_{\mathrm{bcp}}$ typically assumes values around unity in closed-shell interactions [23], including hydrogen bonds [63], whereas much smaller $G/\rho_{\mathrm{bcp}}$ are associated with shared interactions, such as covalent bonds. For halogen bonds, the overall picture seems to be more complicated and intermediate values have been reported [64]. Espinosa et al. [65] have suggested to exploit the ratio |V|/G between the (positive) kinetic energy density and (negative) potential energy density *V* to distinguish between pure closed-shell and incipient shared-shell interactions. With respect to this criterion, all interactions **A** to **D** are associated with values rather close to unity. Only **A**, apparently the strongest XB, can be assigned a significantly negative total energy density *E*.

We are well aware of the fact that much less data for halogen bonds than for hydrogen bonds are available, but attempts have been made to correlate electron density properties in the BCP and interaction energies for XBs [66,67]. Espinosa et al. have established relationships between the potential energy density *V* in the BCP and the interaction energy for hydrogen bonds [36,65]. Strictly speaking, this approach requires careful parametrization for each specific type of contact but it has also been applied to entirely different interactions, e.g., between neighboring azide groups [68]. When we applied the equation originally derived by Espinosa et al. for hydrogen bonds to halogen bonds and tentatively expressed the interaction energy as $E_{\mathrm{XB}}\approx 0.5V_{\mathrm{BCP}}$, the potential energy densities in **1** afforded the interaction energies compiled in Table 2. CrystalExplorer [38] offers an alternative to estimate interaction energies according to benchmarked energy models [39]. In contrast to the approaches above, these interaction energies are not derived from electronic properties at the BCP of the contact atoms only.

Individual components for electrostatic, polarization, dispersion, and repulsion energies thus obtained are compiled in the Supporting Information, and the total interaction energies $E_{\mathrm{tot}}$ for the “fast” energy model are included in Table 2 for comparison with the QTAIM-based approach. In either case, the interactions **C** and **D** occur between the same pair of molecules and have therefore been treated together in Table 2. The most obvious difference between both estimates is encountered for the less conventional π-type interaction, and there might be a good reason: CrystalExplorer, taking all energy contributions between neighboring molecules into account, assigns dispersion energy as dominant for **C** + **D**. In contrast, the approach via $V_{\mathrm{bcp}}$ focuses on specific short contacts and may be better suited for strongly directional interactions limited to just two or perhaps a few contact atoms. Correlation of $V_{\mathrm{bcp}}$ and interaction energies for halogen bonds seems an attractive task for the future.

To the best of our knowledge, no interaction energies have been determined for pyrazole N⋯I halogen bonds. The closest match is the theoretical interaction energy between C_6_F_5_–I⋯pyridine, which amounts to approx. −23.4 kJ mol^−1^ [69]; this value fits well with our estimates for contact **A**. For comparison with **B**, we found the interaction energy of C_6_F_5_–I⋯O=CH_2_ with a value of approximately −19.6 kJ mol^−1^ [70]. In this case, the literature value closely corresponds to the approximation for **B** established by the potential energy density in the BCP. There are not many interaction energies for π⋯I contacts; the closest analogue we found was N≡C–I⋯C_6_H_6_ with about −20.6 kJ mol^−1^ or C_6_H_5_–I⋯C_6_H_6_ with about −12.4 kJ mol^−1^ [54]. No final conclusion can yet be drawn from these values in comparison with contacts **C** + **D**, for which our two estimates also differ, possibly for the reason given above. We want to recall that all these comparisons have to be taken with a grain of salt, mainly for two reasons: (a) the compared data comes from geometrically optimized molecules while we used crystallographic coordinates of **1** for the single-point calculation of ρ and (b) the compared data does not completely match the motif in **1**.

## 4. Conclusions

2,3,5,6-Tetrafluoro-1,4-diiodobenzene is intuitively perceived as a potential bridge between two halogen bond acceptors, and we indeed found this behavior for the shortest and strongest contacts. Competition between two perpendicular TFDIB bonds to the same N acceptor site was much less expected but also encountered in the crystal structure of **1**. This most unusual aspect will most likely also represent the major challenge for future work: How can crystal engineering enhance the frequency of structures in which orthogonal halogen bonds compete for the same acceptor, in our case the iminic N of the pyrazole heterocycle? Once this challenge has been met, fine tuning may target the sequence of the interaction energies and possibly invert the scenario, with stronger I⋯π and weaker I⋯N contacts. The concomitant action of two different XB donor species may provide an additional synthetic degree of freedom for this purpose. We thank one of our reviewers for the following thought-provoking question: Should the three concomitant XBs in **1** be addressed as *competing* or rather as *cooperative* [71]? A competent answer to this question will require more structural input and therefore has to be postponed.

## Figures and Tables

**Table 1:** Short contacts in **1** with properties in their bond critical points (3, −1). BPL is the length of the bond path, ρ the electron density, $\nabla^{2}\rho$ the Laplacian of the electron density, *G* the kinetic, *V* the potential, and *E* the total energy density in the BCP.

Contact	BPL / Å	ρ/eÅ${}^{-3}$	$\nabla^{2}\rho \;/\;e\;$Å${}^{-5}$	*G* / a.u.	G/ρ / a.u.	*V* / a.u.	*E* / a.u.
**A**	2.9120	0.1503	1.4847	0.01635	0.73	−0.01731	−0.00095
**B**	2.9352	0.1093	1.5402	0.01505	0.93	−0.01413	0.00092
**C**	3.2461	0.0746	0.9252	0.00846	0.77	−0.00733	0.00113
**D**	3.2720	0.0217	0.4890	0.00375	1.17	−0.00242	0.00132

**Table 2:** Interaction energies in the non-covalent contacts. $E_{\mathrm{XB}}$ was derived as suggested by Espinosa et al. [36,65] and $E_{\mathrm{tot}}$ was calculated with CrystalExplorer [38,39].

Contact	$E_{\mathrm{XB}}$ / kJ mol${}^{-1}$	$E_{\mathrm{tot}}$ / kJ mol${}^{-1}$
**A**	−22.7	−24.1
**B**	−18.5	−11.5
**C** + **D**	−12.8	−24.9

## Data Availability

CCDC No. 2209103 contains the supplementary crystallographic data for this paper. These data can be obtained free of charge from The Cambridge Crystallographic Data Centre via www.ccdc.cam.ac.uk/data_request/cif (accessed on 25 May 2022).

## Supplementary Materials

The following supporting information can be downloaded at: https://www.mdpi.com/article/10.3390/molecules27217550/s1, Figure S1: Simulated (red) and experimental (black) powder patterns of **1**; Figure S2: Top: Thermogravimetric analysis curve with relative mass loss added. Bottom: Differential scanning calorimetry curve with three integrals added in different colors; Figure S3: Expanded asymmetric unit used for the single-point calculation to derive the electron density ρ in **1**; Figure S4: Trajectory plots in **1** to highlight contacts **A** and **B**; short contacts (hydrogen and halogen bonds) are shown as dashed lines, BCPs (3, −1) as pink spheres, ring critical points (3, 1) as light blue spheres; Figure S5: Trajectory plots in **1** to highlight contacts **C** and **D**; short contacts (hydrogen and halogen bonds) are shown as dashed lines, BCPs (3, −1) as pink spheres, ring critical points (3, 1) as light blue spheres; Table S1: Crystal data for compound **1** at 100(2) K; Table S2: Key data for the differential scanning calorimetry; Table S3: Calculated interaction energies $E_{\mathrm{tot}}$ and their components in kJ mol^−1^ in the non-covalent contacts **A** to **D** calculated with CrystalExplorer; Table S4: Cartesian coordinates of the expanded asymmetric unit (cf. Figure S3) used as Gaussian input for the single-point calculation.

## Abbreviations

The following abbreviations are used in this manuscript:

BCP	bond critical point
BPL	bond path length
HacacMePz	3-(1,3,5-trimethyl-1*H*-4-pyrazolyl)pentane-2,4-dione
Pz	pyrazole
QTAIM	Quantum Theory of Atoms in Molecules
SCXRD	single-crystal X-ray diffraction
TFDIB	2,3,5,6-tetrafluoro-1,4-diiodobenzene
XB	halogen bond
