# Supplementary material for: Three for the Price of One: Concomitant I⋯N, I⋯O, and I⋯π Halogen Bonds in the Same Crystal Structure
Source: Molecules. 2022 Nov 3;27(21):7550. doi: 10.3390/molecules27217550 (PMC9657311; doi:10.3390/molecules27217550)
Supplement: Supplementary file 1 [file molecules-27-07550-s001.zip › molecules-1973062-supplementary.pdf]

Supplementary Materials: Three for the Price of One: Concomitant I···N, I···O and I···π Halogen Bonds in the Same Crystal Structure

Steven van Terwingen [1], Ruimin Wang [1,2] and Ulli Englert [1,2,*]

1. Crystal Data and Refinement Results

Table S1. Crystal data for compound **1** at 100(2) K.

Compound	**1**
Empirical formula	$C_{20}H_{16}F_6I_3N_2O_2$
Moiety formula	$C_{11}H_{16}N_2O_2, 1.5\,(C_6F_4I_2)$
M_r / g mol^{-1}	811.05
Crystal description	colorless block
Crystal size / mm^3	0.16 × 0.16 × 0.12
Crystal system	triclinic
Space group (No.)	$P\bar{1}$ (2)
a / Å	10.4664(18)
b / Å	11.877(2)
c / Å	12.281(2)
α / °	109.054(3)
β / °	103.144(3)
γ / °	111.803(3)
V / Å^3	1229.5(4)
Z	2
D_{calc} / g cm^{-3}	2.191
μ / mm^{-1}	3.879
λ / Å	0.71073
$\sin(\theta_{max})/\lambda$ / Å^{-1}	1.00
total/unique refl.	119971/20085
observed refl.	11830
No. of parameters	306
R_{int}	0.1363
$R(I > 2\sigma(I))$	0.0548
wR_2 (all data)	0.1451
S	1.058
ρ_{min}/ρ_{max} / e Å^{-3}	–2.395/1.901
CCDC #	2209103

2. Powder X-Ray Diffraction

The simulated powder pattern refers to the temperature of the single-crystal diffraction experiment (100 K). As most materials expand with increasing temperature, the simulation is probably associated with a smaller unit cell in real space and thus with larger 2θ in reciprocal space. This results in a slight systematic shift between experimental and simulated diffraction patterns.

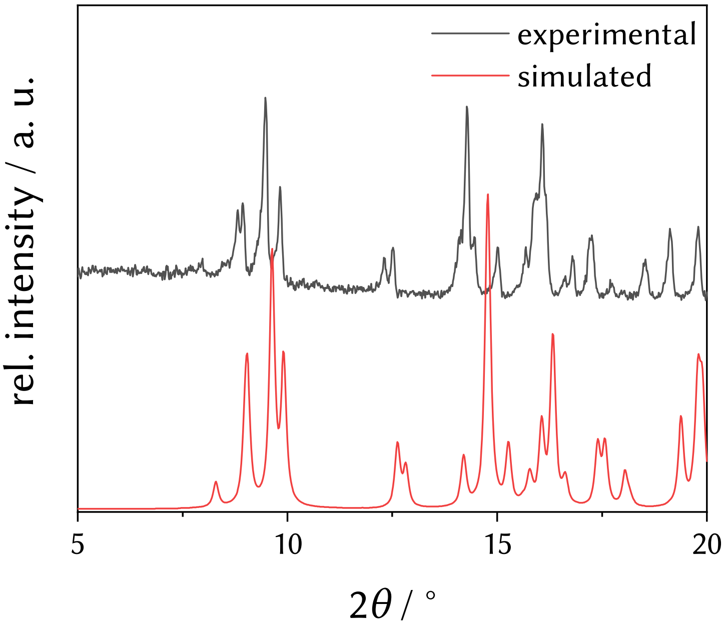

Figure S1. Simulated (red) and experimental (black) powder patterns of **1**.

3. Thermoanalysis

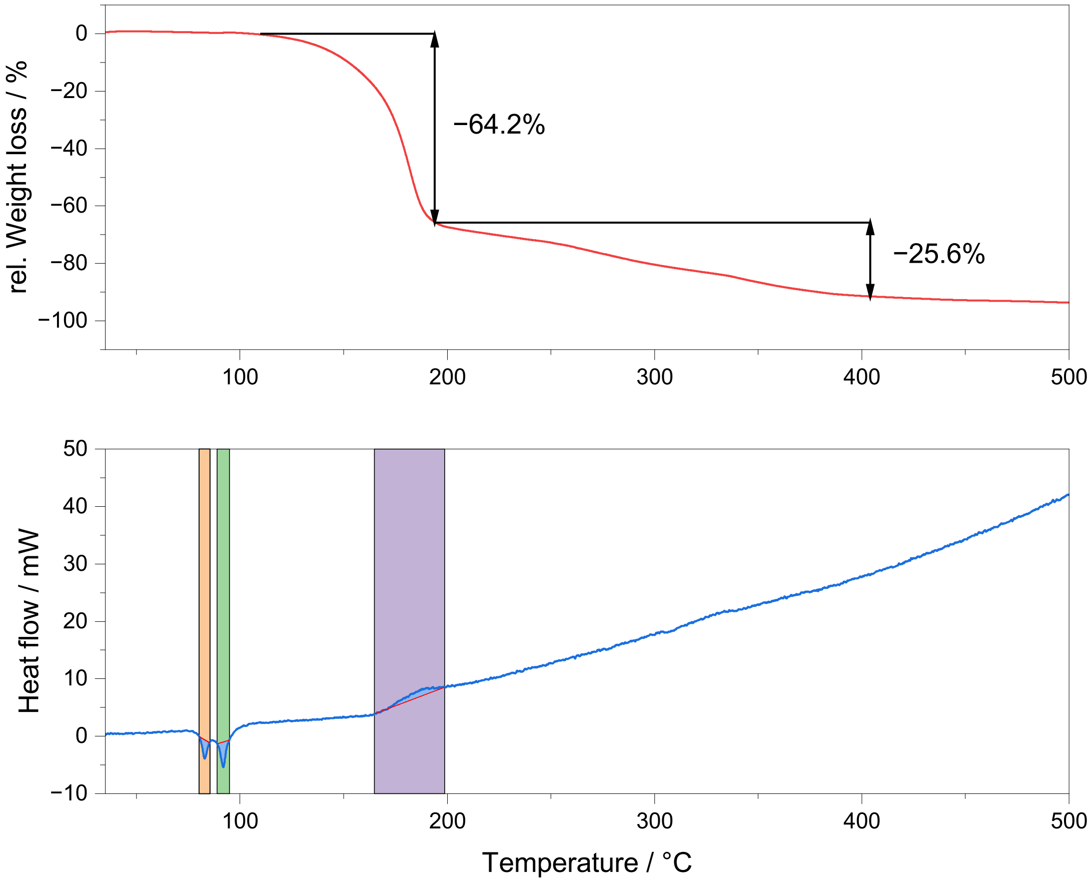

Figure S2. Top: Thermogravimetric analysis curve with relative mass loss added. Bottom: Differential scanning calorimetry curve with three integrals added in different colors.

Table S2. Key data for the differential scanning calorimetry.

Onset / °C	Offset / °C	Maximum / °C	Heat flow / mW	Specific heat capacity / J $K^{-1}g^{-1}$
80.3	85.4	82.8	−3.8	2.8
88.9	94.9	91.9	−5.6	4.2
164.8	198.8	187.8	1.2	/

4. Computational Details

Table S3. Calculated interaction energies E_{tot} and its components in kJ mol^{-1} in the non-covalent contacts **A** to **D** calculated with CrystalExplorer [1,2].

Contact	E_{ele}	E_{pol}	E_{dis}	E_{rep}	E_{tot}
**A**	–44.6	–7.5	–15.5	49.6	–24.1
**B**	–24.7	–3.4	–9.2	29.8	–11.5
**C** + **D**	–20.1	–2.9	–28.9	29.0	–24.9

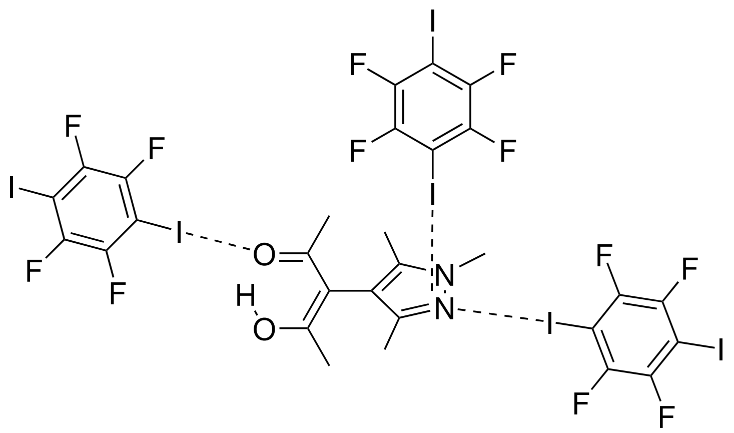

Figure S3. Expanded asymmetric unit used for the single-point calculation to derive the electron density ρ in **1**.

Table S4. Cartesian coordinates of the expanded asymmetric unit (cf. Figure S3) used as Gaussian input for the single-point calculation.

Molecule	Atom type	x	y	z
**HacacMePz**	O	6.215	−0.158	6.138
	O	7.642	1.383	4.844
	N	2.490	1.748	2.150
	N	2.979	0.676	1.470
	C	4.025	−0.772	5.495
	C	5.306	−0.022	5.288
	C	5.465	0.816	4.131
	C	6.662	1.486	3.961
	C	7.017	2.317	2.786
	C	3.134	3.013	4.180
	C	3.330	1.921	3.179
	C	4.360	0.961	3.151
	C	4.088	0.173	2.041
	C	4.812	−1.042	1.554
	C	2.298	0.252	0.261
	H	4.1319	−1.4484	6.3416
	H	3.7926	−1.3457	4.5985
	H	3.2195	−0.0665	5.6935
	H	7.3710	0.8010	5.6015
	H	7.2159	3.3374	3.1100
	H	6.1900	2.3154	2.0773
	H	7.9056	1.9076	2.3072

	H	2.3221	3.6618	3.8559
	H	4.0505	3.5945	4.2665
	H	2.8871	2.5778	5.1476
	H	4.1899	−1.9224	1.7082
	H	5.7433	−1.1557	2.1078
	H	5.0326	−0.9348	0.4932
	H	2.9425	−0.4222	−0.3014
	H	2.0630	1.1225	−0.3493
	H	1.3770	−0.2656	0.5260
**TFDIB A**	I	0.2082	3.3863	1.3921
	F	−2.190	2.890	−0.674
	F	−0.441	6.512	1.802
	C	−2.189	4.181	−0.317
	C	−1.278	4.646	0.616
	C	−1.310	5.995	0.919
	I	−4.6195	7.6411	−1.3921
	F	−2.221	8.137	0.674
	F	−3.970	4.516	−1.802
	C	−2.222	6.846	0.317
	C	−3.133	6.381	−0.616
	C	−3.101	5.032	−0.919
**TFDIB B**	I	8.3471	−0.3805	8.1340
	F	10.778	−2.182	9.160
	F	8.884	2.028	10.164
	C	10.739	−1.048	9.878
	C	9.780	−0.092	9.587
	C	9.775	1.055	10.388
	I	13.0554	0.5373	13.1715
	F	10.624	2.338	12.145
	F	12.519	−1.872	11.142
	C	10.664	1.205	11.427
	C	11.623	0.248	11.718
	C	11.628	−0.898	10.918
**TFDIB C**	I	0.4654	−0.2993	3.6371
	F	−2.568	−1.254	3.364
	F	1.209	−2.352	5.993
	C	−1.973	−1.959	4.339
	C	−0.638	−1.745	4.640
	C	−0.067	−2.527	5.648
	I	−3.2581	−5.1360	7.0157
	F	−0.225	−4.181	7.289
	F	−4.002	−3.083	4.660
	C	−0.820	−3.476	6.314
	C	−2.155	−3.691	6.012
	C	−2.725	−2.909	5.005

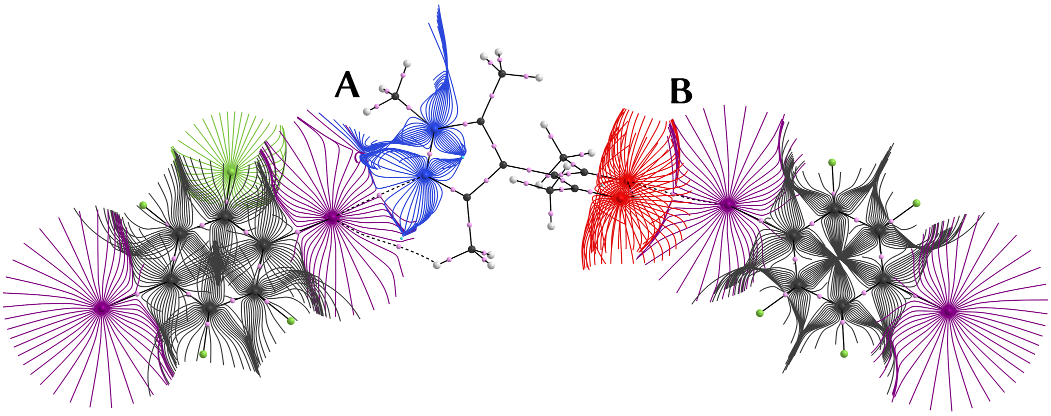

Figure S4. Trajectory plots [3] in **1** to highlight contacts **A** and **B**; short contacts (hydrogen and halogen bonds) are shown as dashed lines, BCPs (3, –1) as pink spheres, ring critical points (3, 1) as light blue spheres.

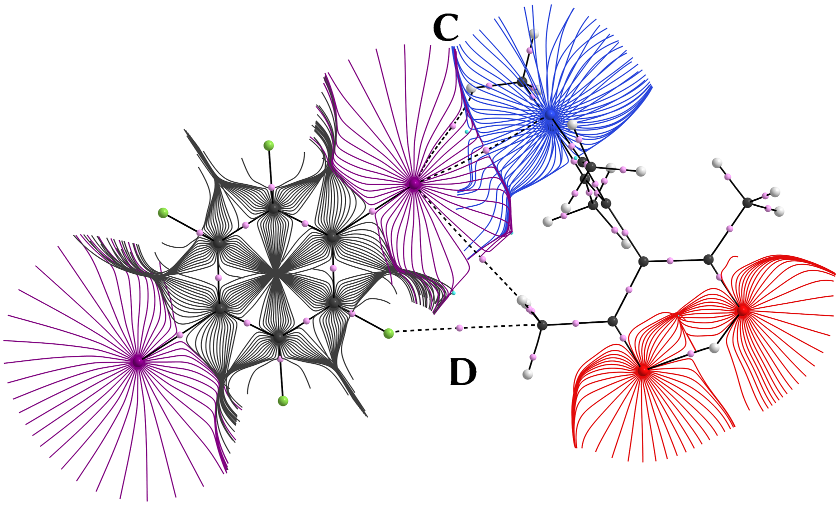

Figure S5. Trajectory plots [3] in **1** to highlight contacts **C** and **D**; short contacts (hydrogen and halogen bonds) are shown as dashed lines, BCPs (3, –1) as pink spheres, ring critical points (3, 1) as light blue spheres.

References

1. Spackman, P.R.; Turner, M.J.; McKinnon, J.J.; Wolff, S.K.; Grimwood, D.J.; Jayatilaka, D.; Spackman, M.A. CrystalExplorer: a program for Hirshfeld surface analysis, visualization and quantitative analysis of molecular crystals. *J. Appl. Crystallogr.* **2021**, *54*, 1006–1011. https://doi.org/10.1107/S1600576721002910.
2. Turner, M.J.; Grabowsky, S.; Jayatilaka, D.; Spackman, M.A. Accurate and Efficient Model Energies for Exploring Intermolecular Interactions in Molecular Crystals. *J. Phys. Chem. Lett.* **2014**, *5*, 4249–4255. https://doi.org/10.1021/jz502271c.
3. Keith, T.A. *AIMAll: Version 17.01.25*; TK Gristmill Software: Overland Park KS, USA, 2017.
